# Coexistence of Autoimmune Thyroiditis and Juvenile Idiopathic Arthritis

**DOI:** 10.7759/cureus.44384

**Published:** 2023-08-30

**Authors:** Evdoxia Sapountzi, Vasiliki-Rengina Tsinopoulou, Eleni P Kotanidou, Styliani Giza, Assimina Galli-Tsinopoulou

**Affiliations:** 1 2nd Department of Pediatrics, Aristotle University of Thessaloniki, AHEPA University General Hospital, Thessaloniki, GRC

**Keywords:** thyroglobulin, thyroid peroxidase, hashimoto, autoimmune thyroiditis, juvenile idiopathic arthritis

## Abstract

Purpose: Autoimmune thyroid disease seems to occur more often in children with juvenile idiopathic arthritis (JIA) than in the general pediatric population. We investigated the prevalence of autoimmune thyroiditis (Hashimoto’s thyroiditis) in young patients with JIA in Greece, which has not been evaluated previously.

Methods: This descriptive study included patients with JIA followed up at the Pediatric Rheumatology Unit of the Second Department of Pediatrics of a tertiary general hospital in Thessaloniki, Greece. All patients were diagnosed and sorted according to the classification criteria of the International League of Associations for Rheumatology. The presence of thyroid autoantibodies was considered for determining autoimmune thyroiditis. Basic demographic, clinical, and laboratory data were collected from patients’ records.

Results: The analyzed sample comprised 130 patients with JIA (mean age 12.22 years; 69.2% female). Most patients (70%) had a family history of at least one autoimmune disease and 30.8% of Hashimoto’s thyroiditis. More than half (53.8%) had enthesitis-related arthritis (ERA), 22.3% had oligoarthritis, and 15.4% had psoriatic arthritis. Thyroid autoantibodies were detected in 22/130 patients (16.9%) suggesting autoimmune thyroiditis; most of these patients were euthyroid, whereas 3/22 (13.6%) had overt hypothyroidism determined by elevated levels of thyroid-stimulating hormone, decreased levels of free thyroxine, and typical ultrasound findings for Hashimoto's thyroiditis. The prevalence of clinical cases of Hashimoto’s disease was 2.3%.

Conclusions: The prevalence of autoimmune thyroiditis in our JIA cohort is higher compared to the general population and consistent with the previously reported range. Hence, investigation for thyroid autoimmunity should be included in the workup of patients with JIA.

## Introduction

Autoimmune diseases constitute a highly prevalent disease group. To date, more than 100 autoimmune disorders have been described, many of which share common characteristics in terms of pathogenic mechanisms and may coexist with them [1]. Autoimmune thyroid diseases include several inflammatory thyroid disorders, the most frequent of which are Hashimoto’s thyroiditis and Graves' disease. Hashimoto’s thyroiditis, also referred to as chronic autoimmune thyroiditis and autoimmune hypothyroidism has a global prevalence of 7.5%, affecting females more than males (prevalence of 17.5% in females and 6% in males) [2]. In children and adolescents, Hashimoto’s thyroiditis is the most common cause of thyroid disease, with a prevalence of approximately 3% [3].

The diagnosis of autoimmune thyroiditis is based on the presence of thyroid autoantibodies, as well as on sonographic abnormalities in the thyroid gland, consistent with lymphocytic infiltration [4]. However, autoantibody levels rise before ultrasonographic findings and are detected well before symptoms appear [5]. Hence, their detection may help predict patients at risk of developing hypothyroidism and thus provide the best treatment and follow-up options. Importantly, it was previously shown that metal or vitamin supplementation reduces the levels of anti-thyroid antibodies in patients with autoimmune thyroiditis, which may stall the progression of thyroid gland destruction [6, 7].

There is some evidence that autoimmune thyroid disease occurs more often in children with juvenile idiopathic arthritis (JIA) than in the general pediatric population [8-11]. JIA is the most common pediatric rheumatological disorder and constitutes an umbrella harboring heterogeneous diseases with multifactorial and different pathogenesis [12]. It affects children up to 18 years of age and its global prevalence ranges from 3.8 to 400 per 100,000, while its incidence ranges from 1.6 to 23 per 100,000 children, with girls being more affected than boys [13,14]. JIA has been shown to coexist with other autoimmune disorders [1,15], although this has been controversial with different studies reaching different conclusions, which has resulted in a lack of screening tests in routine patient examinations [16].

Interestingly, there are several reports of concurrent JIA with autoimmune thyroiditis, while some case series have shown a high prevalence of thyroid autoantibodies in JIA, particularly in patients with oligoarticular disease [11]. However, these existing studies are limited to case reports or small sample studies, and, to our knowledge, no study has described the coexistence of Hashimoto’s thyroiditis in patients with JIA in Greece. Hence, we aimed to investigate the prevalence of Hashimoto’s thyroiditis in young patients with JIA in a single tertiary center in Greece.

## Materials and methods

Study design

This was an observational cross-sectional study conducted using the medical data of patients followed-up at the tertiary Pediatric Rheumatology Unit of the 2nd Department of Pediatrics at AHEPA University General Hospital in Thessaloniki, the second largest city in Greece, from January 1, 2017, to October 1, 2022.

This study was conducted in accordance with the principles of the Declaration of Helsinki, and the protocol is part of a broad study of Hashimoto's thyroiditis in children and adolescents, which was submitted and approved by the Ethics Committee of the School of Medicine, Faculty of Health Sciences, Aristotle University of Thessaloniki (approval number: 1/18.12.2013). The enrolled patients voluntarily agreed to participate in the study, and written informed consent was obtained from their parents or guardians. 

Patients

Children and adolescents aged <18 years who had at least one outpatient clinic visit or at least one hospital admission were included. The JIA cohort comprised individuals who had instances of diagnoses coded for JIA using the International Classification of Diseases, 10th Revision, Clinical Modification (ICD-10-CM) system. The date of diagnosis of JIA was recorded as the time of patient encounter or the date of discharge diagnosis. If there were multiple dates, we incorporated their more recent visits to prevent any potential misdiagnosis. All patients were diagnosed and classified based on the latest consensus regarding JIA definition and classification criteria [14]. 

Patients with an unclassified JIA subtype or those who were not screened for autoimmune thyroid disease were not included in the analysis. Furthermore, patients were excluded if they had any of the following conditions: Raynaud syndrome, chronic glomerulonephritis, myositis, Sjogren syndrome, or interstitial lung disease, i.e., conditions that are commonly present in other rheumatic diseases and may have led to an initial misdiagnosis of JIA. To confirm the presence of autoimmune thyroid disease, the ICD‑10-CM diagnosis code for Hashimoto’s disease was used.

Basic demographic, clinical, and laboratory data were collected from patients’ records at the first presentation and at follow-up sessions. Specifically, the following parameters were recorded: age, sex, family history of autoimmune disease, family history of Hashimoto’s thyroiditis, comorbidities, presence of thyroid autoantibodies (anti‑thyroid peroxidase (TPO) and/or anti‑thyroglobulin (TG)), thyroid stimulating hormone (TSH) and free thyroxine (fT4) levels, presence of antinuclear antibodies (ANA) and human leukocyte antigen B27 (HLA-B27), and vitamin D profile. In detail, serum samples were used to measure the levels of fT4, TSH, and antibodies to TPO and TG by chemiluminescence immunoassay (Immulite® 2000 Advanced Immunoassay System, Diagnostic Products Corporation, Los Angeles, California, United States).

Reference ranges for thyroid markers are subject to the laboratory where the test is performed. The cut-off point for seropositivity of anti-TPO and anti-TG titers was 60 IU/mL. Patients with clinical or laboratory hyperthyroidism were excluded. Thyroid gland ultrasound was performed in all patients with suspected Hashimoto's thyroiditis by the same experienced radiologist using a Sonoline G50 ultrasound system (Siemens Healthineers). Hashimoto’s disease was considered if patients tested positive for anti-TPO and/or anti-TG antibodies. Vitamin 25-hydroxy vitamin D (25(OH)D) levels were measured in serum via the cobas e 602 immunochemistry module using electrochemiluminescence technology (F. Hoffmann-La Roche AG, Basel, Switzerland). Vitamin D deficiency was determined if levels were ≤20 ng/mL [7].

Data analysis

Continuous data were presented as mean and standard deviation of the mean, whereas qualitative data were described as frequencies (%). The normality of the distribution of continuous variables was tested by Kolmogorov-Smirnov or Shapiro-Wilk test, as appropriate. Comparisons of continuous data distribution were performed with t-test or its non-parametric analog Mann Whitney U test. Categorical variables were compared using chi-square test. The level of statistical significance was defined at p < 0.05. Statistical analysis was performed using IBM SPSS Statistics for Windows, Version 25.0 (Released 2017; IBM Corp., Armonk, New York, United States).

## Results

A total of 132 patients were enrolled in this study. Two of these patients were excluded as they were diagnosed with unclassified or undetermined JIA according to the inclusion/exclusion criteria. Thus, the analyzed sample comprised 130 patients with JIA. Patient characteristics are presented in Table 1.

**Table 1:** Patient characteristics Categorical variables are presented as frequency (%). Numerical variables are presented as mean (SD). P-values in bold denote statistically significant differences between the Hashimoto and non-Hashimoto groups (<0.05). ANA, antinuclear antibodies; fT4, free thyroxine; HLA-B27, human leukocyte antigen B27; IBD, inflammatory bowel disease; JIA, juvenile idiopathic arthritis; RF, rheumatoid factor; SD, standard deviation; TG, thyroglobulin; TPO, thyroid peroxidase; TSH, thyroid stimulating hormone.

Variable	Total (N=130)	Hashimoto’s thyroiditis (N=22)	no Hashimoto’s thyroiditis (N=108)	p-value
	Mean ± SD or n (%)			
Age (years)	12.22 ± 3.50	13.14 ± 3.48	12.04 ± 3.49	0.197
Sex (Female)	90 (69.2)	18 (81.8)	70 (64.8)	0.120
Family history of autoimmune disease	91 (70.0)	16 (72.7)	76 (70.4)	
1 autoimmune disease	30 (23.1)	4 (18.2)	26 (24.1)	0.550
2 autoimmune diseases	36 (27.7)	5 (22.7)	31 (28.7)	0.568
>2 autoimmune diseases	25 (19.2)	6 (27.3)	19 (17.6)	0.294
Family history of Hashimoto’s thyroiditis	40 (30.8)	7 (31.8)	33 (30.6)	0.907
JIA category				0.421
Enthesitis-related	70 (53.8)	16 (72.7)	54 (50.0)	
Oligoarticular	29 (22.3)	2 (9.1)	27 (25.0)	
Psoriatic	20 (15.4)	2 (9.1)	18 (16.7)	
Polyarticular	9 (6.9)	2 (9.1)	7 (6.5)	
RF-	7 (5.4)	2 (9.1)	5 (4.6)	
RF+	2 (1.5)	0 (0.0)	2 (1.9)	
Systemic	1 (0.8)	0 (0.0)	1 (0.9)	
with IBD	1 (0.8)	0 (0.0)	1 (0.9)	
Anti-TPO positive only	5 (3.8)	5 (22.7)	0 (0.0)	<0.001
Anti-TG positive only	7 (5.4)	7 (31.8)	0 (0.0)	<0.001
Anti-TPO and Anti-TG positive	10 (7.7)	10 (45.5)	0 (0.0)	<0.001
ANA positive	65 (50.0)	12 (54.5)	53 (49.1)	0.640
1/160	38 (29.2)	6 (27.3)	32 (29.6)	
<1/160	27 (20.7)	6 (27.2)	21 (19.4)	
HLA-B27 positive	18 (13.8)	5 (22.7)	13 (12.0)	0.186
TSH (mIU/L)	2.4 (1.2)	3.1 (1.7)	2.3 (0.9)	0.016
TSH, abnormal	6 (4.6)	5 (22.7)	1 (0.9)	<0.001
high	5 (3.8)	4 (18.2)	1 (0.9)	
fT4 (ng/dL)	2.86 (4.3)	3.5 (4.6)	2.7 (4.3)	0.296
fT4, low	1 (0.8)	1 (4.5)	0 (0.0)	0.026
Vitamin D (ng/mL)	27.91 ± 11.57	27.55 ± 17.09	27.99 ± 10.20	0.216
10-20 (Vitamin D deficiency)	29 (22.3)	7 (31.8)	22 (20.4)	
>20	101 (77.7)	15 (68.2)	86 (79.6)	

The mean age of patients was 12.22 ± 3.50 years, and 69.2% were female. The majority of patients (70%) had a family history of at least one autoimmune disease, and 30.8% had a family history of Hashimoto’s thyroiditis. 

With regards to JIA classification according to Paediatric Rheumatology International Trials Organisation (PRINTO) criteria, most patients (53.8%) had enthesitis-related arthritis (ERA), followed by patients with oligoarthritis (22.3%) and those with psoriatic arthritis (15.4%). Only nine patients (6.9%) had polyarthritis, with seven (5.3%) testing negative for rheumatoid factor and two (1.5%) testing positive. Systematic arthritis was found only in one patient, and one patient had JIA with inflammatory bowel disease. 

Half of the patients (N=65, 50%) tested positive for antinuclear antibody (ANA) which may suggest the presence of an autoimmune disease. Strong ANA positivity (1/1280) was detected in 3.1% of the patients, with half of them exhibiting a homogenous pattern and the remaining showing a speckled pattern. Moreover, 13.8% of the patients were positive for human leukocyte antigen (HLA)-B27, while 22.3% had vitamin D deficiency (levels ranging between 10 and 20 ng/mL).

According to the presence of thyroid autoantibodies (anti-TPO and/or anti-TG), Hashimoto’s thyroiditis was diagnosed in 22 patients (16.9%), the majority of whom were euthyroid, while three had overt hypothyroidism determined by elevated levels of TSH, decreased levels of fT4, and typical ultrasound findings for Hashimoto's thyroiditis: a slightly enlarged thyroid gland with a mildly heterogeneous echotexture. The vast majority of patients with thyroid autoantibodies (81.8%) were females. The most prevalent JIA subtype amongst them was ERA (16 of 22 patients; 72.7%). Almost 32% of the patients with Hashimoto’s thyroiditis had vitamin D deficiency. Among patients positive for anti-TPO (N=5, 3.8%; all females), only two presented elevated TSH levels. Of those, one had low levels of fT4, indicating a case of primary hypothyroidism; this patient was negative for ANA and HLA‑B27. The second patient was positive for ANA and HLA‑B27. Seven patients were tested positive for anti‑TG (5.4%; four females/three males). All of these individuals had normal levels of TSH and fT4 except for one who exhibited hypothyroidism. Ten patients (7.7%; nine females/one male) were positive for both thyroid autoantibodies, of whom eight were also positive for ANA, and two for HLA-B27. Of these 10 patients, two presented elevated TSH levels (one with hypothyroidism) and one had decreased levels. 

The only significant differences between patients with and those without Hashimoto’s thyroiditis were in TSH levels and in the percent of patients with abnormal TSH and fT4 levels (Table 1). 

## Discussion

The main finding of the present study was that Hashimoto's autoimmune thyroiditis is a common (16.9%) autoimmune disorder in children and adolescents with JIA.

Previous studies have demonstrated variable results regarding the frequency of autoimmune thyroiditis among children with autoimmune diseases, ranging between 5% and 44% [17-20]. The prevalence in the present study was 16.9%, which is within this range and more than double the estimated global prevalence of Hashimoto’s disease of 7.5% [2]. Among females, the prevalence was even higher, 20%, which is again higher than the global prevalence in females of 17.5% [2].

Straalen et al. reported a prevalence of Hashimoto’s thyroiditis of less than 1% in patients with JIA [21]. This study marked the primary instance of identifying distinct predictors for autoimmune thyroid disease in JIA, utilizing data sourced from the worldwide observational PharmaChild Registry. This much lower rate can be justified by the fact that the authors did not consider laboratory parameters for diagnosing autoimmune thyroiditis and only considered clinical cases, i.e., only the following MedDRA (Medical Dictionary for Regulatory Activities) preferred terms were considered for determining autoimmune thyroiditis: “hypothyroidism”, “autoimmune thyroiditis”, “thyroiditis”, “hyperthyroidism”, and “Basedow’s disease”.

Similar to previous studies, our study was more inclusive, also considering subclinical cases. This is important since anti‑thyroid antibodies are detected well ahead of the manifestation of hypothyroidism [5], and early diagnosis facilitates improved follow-up and early detection of disease activity, enabling the early implementation of treatment to prevent cellular destruction and the development of goiter. When only considering clinical cases from our cohort, there were three patients with overt hypothyroidism, leading to a prevalence of 2.3%, which is still much higher than that reported by Straalen et al. (0.65%) [21]. However, the cost-effectiveness of screening all these patients should be investigated further before we change our practice. Notably, the percentage of patients with a family history of autoimmune disease and that of patients with a family history of Hashimoto’s thyroiditis in our study (70% and 30.8%, respectively) were much higher than that in the study by Straalen et al. (30.4% and 5.1%, respectively) [21], which may explain the different findings of the two studies. 

Most patients with Hashimoto’s thyroiditis in our study suffered from enthesitis-related JIA (72.7%) contrary to previous reports that mentioned a higher occurrence of autoimmune thyroiditis/thyroid autoantibodies among patients with oligoarticular disease [11,18, 21]. This could be explained by the overall higher occurrence of ERA in our cohort (53.8%) compared with the known occurrence of ERA among patients with JIA (15%-20%) [22]. Another study by Tronconi et al. demonstrated no correlation between autoimmune thyroiditis and any specific JIA subtype [20]. These equivocal findings underscore the need for additional investigations, highlighting the ambiguous nature of these results and the necessity for further studies. 

In the present study, approximately 32% of the patients with Hashimoto’s thyroiditis had vitamin D deficiency. This percentage was higher than that among patients without Hashimoto’s (around 20%). This result is consistent with the negative correlation between vitamin D levels and the levels of anti-thyroid antibodies both in adult and pediatric patients with Hashimoto’s thyroiditis [7]. 

Our results should be interpreted considering certain limitations. First, we determined the prevalence of Hashimoto’s thyroiditis merely on the presence of thyroid autoantibodies. Although these are present in more than 90% of the patients with Hashimoto’s, there are 10% of the patients who test negative [23]. Patients with other types of autoimmune thyroid diseases may also have elevated anti-TPO and/or anti-TG antibodies [24]. Hence, the calculated prevalence may have been either under- or overestimated. Second, our analyzed sample was relatively small, comprising 130 patients with JIA. Although epidemiological data for JIA are not available in Greece, our sample is justified when considering the incidence of the disease. Finally, this study was conducted in a single center and only provided descriptive results since it did not include a control sample. Hence, further larger sample, multi-center, controlled studies are needed to confirm the present findings. 

## Conclusions

Our study reveals the presence of subclinical autoimmune thyroiditis in around 17% of patients with JIA in a tertiary center in Greece. The prevalence of clinical cases was 2.3%. Both findings are within the ranges published previously. Considering that the symptoms of Hashimoto’s thyroiditis might appear late in the progression of the disease and that the disease might remain undiagnosed for years, we propose that screening for thyroid antibodies may be included in the examinations panel for patients with JIA.
